# CRISPR-Cas13a-based detection method for avian influenza virus

**DOI:** 10.3389/fmicb.2023.1288951

**Published:** 2023-10-11

**Authors:** Yuhan Wu, Jiaxing Zhan, Zhaomeng Shan, Yanbing Li, Yining Liu, Yan Li, Yixin Wang, Zhe Liu, Xuexia Wen, Xiurong Wang

**Affiliations:** ^1^Key Laboratory of Livestock Infectious Diseases, Ministry of Education, College of Animal Science and Veterinary Medicine, Shenyang Agricultural University, Shenyang, China; ^2^State Key Laboratory for Animal Disease Control and Prevention, Harbin Veterinary Research Institute, Chinese Academy of Agricultural Sciences, Harbin, China

**Keywords:** avian influenza virus, recombinase polymerase amplification, CRISPR-cas13a, lateral flow dipstick assay, universal detection

## Abstract

Avian influenza virus (AIV) causes huge losses to the global poultry industry and poses a threat to humans and other mammals. Fast, sensitive, and portable diagnostic methods are essential for efficient avian influenza control. Here, a clustered regularly interspaced short palindromic repeats (CRISPR)-Cas13a based platform was developed to detect AIV. This novel method was developed to specifically detect H1–H16 subtypes of AIV with fluorescence and lateral flow-based readouts and exhibited no cross-reactivity with Newcastle disease virus, avian infectious bronchitis virus, or infectious bursal disease virus. The limit of detection was determined to be 69 and 690 copies/μL using fluorescence and lateral flow as readouts, respectively. The developed assay exhibited 100% consistency with quantitative real-time polymerase chain reaction in detecting clinical samples. The heating of unextracted diagnostic samples to obliterate nuclease treatment was introduced to detect viral RNA without nucleic acid extraction. Single-step optimization was used to perform reverse transcription, recombinase polymerase amplification, and CRISPR-Cas13a detection in a tube. These advances resulted in an optimized assay that could specifically detect AIV with simplified procedures and reduced contamination risk, highlighting the potential to be used in point-of-care testing.

## 1. Introduction

Avian influenza (AI) was a viral infectious disease caused by the avian influenza virus (AIV), of the *Orthomyxoviridae* family (Spackman, 2014). Based on the pathogenicity of experimentally infected chickens, AIVs were classified into two categories: low and highly pathogenic avian influenza viruses (LPAIV and HPAIV, respectively). LPAIVs caused either no signs of disease or mild disease, such as lower egg production or respiratory symptoms, whereas HPAIV infection caused severe disease and high mortality (Tian et al., 2023).

AIVs are enveloped negative-stranded RNA viruses (Yamayoshi et al., 2016). Their genome contains 8 separate RNA segments: basic polymerase 2 (PB2), basic polymerase 1 (PB1), acidic polymerase (PA), hemagglutinin (HA), nucleoprotein, neuraminidase (NA), matrix (M), and nonstructural protein, which encode 10 essential proteins. Based on the antigenicity of the two surface glycoproteins, HA and NA, AIVs were categorized into 16 different HA subtypes and 9 different NA subtypes in poultry or wild birds (Fouchier et al., 2005; Tong et al., 2012, 2013). Currently, only strains bearing the HA gene of the H5 and H7 subtypes have proven to be highly pathogenic in naturally infected poultry (Li et al., 2014; Zeng et al., 2020; Li and Chen, 2021).

AIVs have decimated the global poultry industry and were serious threats to humans and other mammals (Zhang et al., 2013; Cui et al., 2022). Human AIV infection was first reported in Hong Kong in 1997, and subsequently several human infection cases, including fatal ones, had occurred (Taubenberger and Kash, 2010; World Health Organization, 2023). Therefore, control of AI is essential for both the poultry industry and public health. Developing fast, sensitive, and portable diagnostic methods is important for controlling AI.

Cas13a is an RNA-guided ribonuclease that recognizes its target RNA through base pairing with clustered regularly interspaced short palindromic repeat (CRISPR)-derived RNA (crRNA) (Gootenberg et al., 2017). After recognition and cleavage of the RNA target, Cas13a remains active and can cleave non-target RNA sequences (East-Seletsky et al., 2016). Fluorescent reporter and quenching groups are labeled at the 5′ and 3′ ends of the non-target RNA sequences, respectively; as the non-target RNA is cleaved, fluorescence is detectable. Therefore, the presence or absence of fluorescence could be used to determine whether Cas13a specifically recognized its target RNA through crRNA (Zhao et al., 2022).

Recombinase polymerase amplification (RPA) technology could amplify nucleic acid at a constant temperature and is highly sensitive, efficient, and cost-effective (Qian et al., 2020). Compared with PCR technology, RPA can be performed at a constant temperature without complicated operations and with low requirements for personnel and equipment, making it suitable for diagnosis at the grassroots or on-site level (Chen et al., 2021). However, RPA is susceptible to non-specific amplification at low template concentrations or in the absence of a template because it operates under isothermal conditions. The ability of Cas13a to specifically recognize target RNA through crRNA indicates it could also be used to improve the specificity of RPA technology. Studies have shown that combining the CRISPR-Cas system with RPA technology for pathogen detection can increase sensitivity by up to a million-fold (Gootenberg et al., 2017).

**Figure 1:**
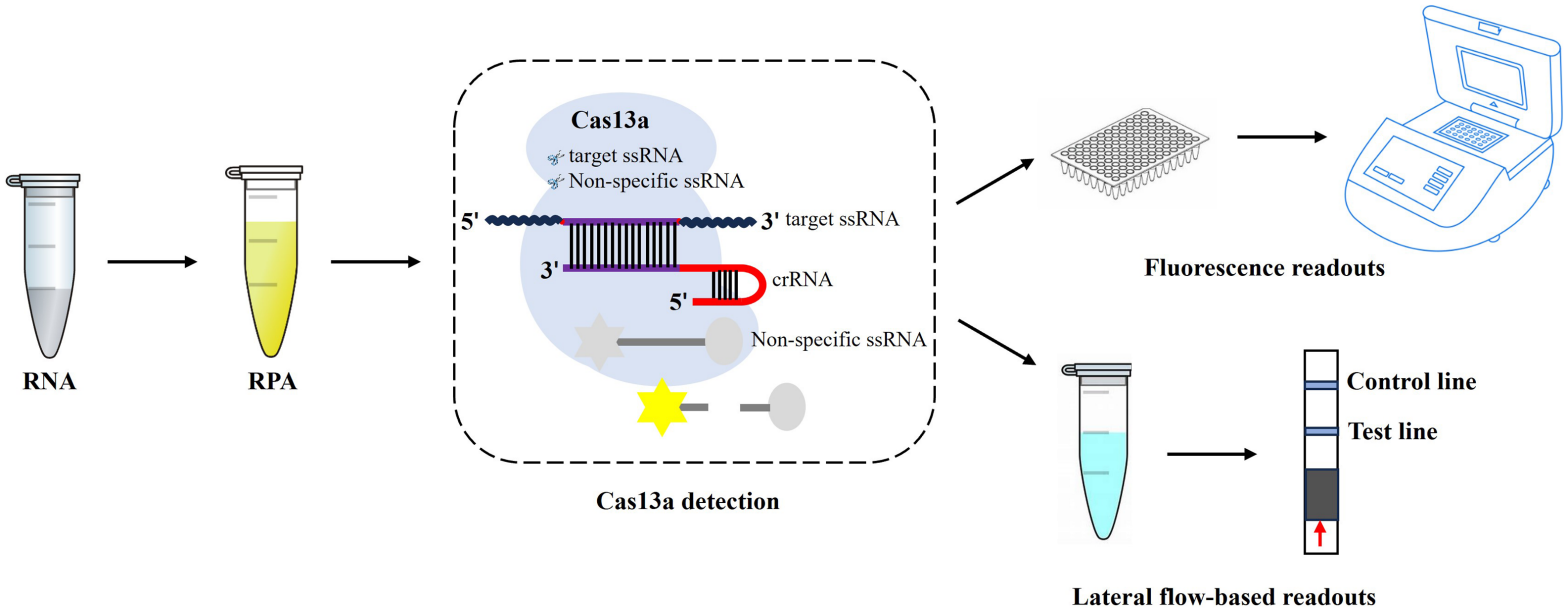
Workflow of CRISPR-Cas13a-based detection of AIV. The AIV RNA is extracted, which is then used for RPA amplification, and followed by CRISPR-Cas13a detection. After recognition and cleavage of the specific RNA target, Cas13a remains the “collateral activity” and cleave bystander non-specific fluorescently labeled ssRNA to produce fluorescent signals. The released fluorescent signals can be detected by fluorescence readers or lateral flow strips.

The M segment is relatively conserved and is considered an attractive region for AIV detection (Shi et al., 2019). In this study, we aimed to combine the CRISPR-Cas13a system with RPA technology in an effort to establish a novel method for the universal detection of all subtypes of AIV (Figure 1). This work could provide a promising platform for diagnosing and monitoring AI.

## 2. Materials and methods

### 2.1. Viruses and clinical samples

H1–H16 reference influenza viruses were stored in the National Avian Influenza Reference Laboratory at the Harbin Veterinary Research Institute (HVRI) of the Chinese Academy of Agricultural Sciences (CAAS) (Table 1). Newcastle disease virus (NDV), avian infectious bronchitis virus (IBV), and infectious bursal disease virus (IBDV) samples (Table 1) were obtained from research teams at the State Key Laboratory of Veterinary Biotechnology of HVRI, CAAS. Thirty clinical samples including 24 swab samples, and 6 lung tissue samples were collected from 2016 through 2019 in Liaoning province from chickens with suspected AIV clinical signs, including tearing, coughing, gasping, diarrhea, and significant drops in egg production.

**Table 1:** Reference strains used in this study.

Name of reference strains	Subtype
A/mallard/Sanjiang/390/2007	HIN1
A/mallard/Heilongjiang/135/2006	H2N2
A/mallard/Heilongjiang/90/2006	H3N2
A/duck/Guangxi/S-2-248/2009	H4N6
A/Chiken/HuN/1/2016	H5N6
A/mallard/Heilongjiang/81/2006	H6N2
A/pigeon/Shanghai/S1069/2013	H7N9
A/turkey/ontario/6118/1968	H8N4
A/Turkey/Wisconsin/1/66	H9N2
A/Turkey/England/384/1979	H10N4
A/Duck/Memphis/546/1976	H11N9
A/duck/Alberta/60/1976	H12N5
A/gull/Maryland/704/1977	H13N6
A/mallard/Gurjer/263/1982	H14N5
A/duck/Australia/341/83	H15N8
A/cormorant/Denmark/74-68899-G2/02	H16N3
NDV	LaSota
IBV	Lx4
IBDV	Gt

### 2.2. cDNA preparation

The lung tissue samples were added to 1.5 mL tubes containing sterilized phosphate-buffered saline (PBS) and steel balls. These samples were then homogenized in a grinder (DHS Life Science & Technology Co., Ltd., Beijing, China) for 30 s at 60 Hz and 4°C. Following this, the tissue homogenates were freeze-thawed thrice and then centrifuged at 12,000 × g at 4°C for 1 min. The supernatants were kept for nucleic acid extraction.

TRIzol (Magen Biotechnology, Guangzhou, China) was used to extract viral nucleic acids. Briefly, 250 μL of each supernatant sample was lysed with 750 μL of TRIzol, followed by the addition of 250 μL of chloroform to allow the RNA to enter the aqueous phase. Subsequently, an equal volume of isopropanol was used to precipitate the RNA. After two washes with 75% ethanol, 30 μL nuclease-free water was added to dissolve RNA, which was then stored at −80°C for further experiments. cDNAs were prepared using a reverse transcription kit (Nanjing Vazyme Biotech Co., Ltd. Nanjing, China) with the extracted RNA following the manufacturer’s instructions.

### 2.3. RPA assay

RPA primers were designed based on the highly conserved regions of the AIV M segment; the sequences used are shown in Table 2. RPA amplification was performed using an RPA amplification kit (TwistDX, Maidenhead, England) in a 50 μL reaction volume, including 29.5 μL of primer-free rehydration buffer, 11.2 μL of nuclease-free water, 2.4 μL of each primer, 2 μL of sample cDNA, and 2.5 μL of 280 mM magnesium acetate (MgOAc). A total volume of 47.5 μL of rehydration solution containing primers and template RNA was used to resuspend the freeze-dried reaction pellets. Then, 2.5 μL of 280 mM MgOAc was added to initiate the reaction. The reaction was performed in an incubator block for 20 min at 39°C. After 4 min of incubation, the tubes were removed, vortexed, spun down briefly, and returned to the incubator block for the remaining 16 min.

**Table 2:** Sequences of primers and crRNA.

Name	Sequence (5′ → 3′)
M-RPA-F	CTCACCGTGCCCAGTGAGCGAGGACTGCAG
M-RPA-R	CCATCCTATTGTATATGAGGCCCATGCAAC
crRNA	GAUUUAGACUACCCCAAAAACGAAGGGGACUAA
	AACAUGGAAAUGGGGACCCGAACAACAUGGA

### 2.4. Preparation and purification of crRNA

The crRNA was designed according to the characteristics of the *Lwa*Cas13a enzyme. The sequences are shown in Table 2. The single-stranded DNA oligonucleotides used for crRNA preparation were synthesized and diluted to a concentration of 50 μM with nuclease-free water. Subsequently, the single-stranded DNA oligonucleotides were annealed using DNA annealing buffer (Beyond Biotechnology Co., Ltd., Shanghai, China) to form double-stranded DNA. Annealing was carried out in a 100 μL reaction volume, including 40 μL of nuclease-free water, 20 μL of annealing buffer (5×), and 20 μL of forward or reverse oligonucleotide. Each oligonucleotide pair was annealed at 95°C for 2 min, followed by a shift in the temperature gradient down to 25°C within 2 h.

Double-stranded DNA templates were transcribed *in vitro* using a T7 RNA Synthesis Kit (New England Biolabs, Ipswich, MA, United States). The transcription was performed in a 30 μL cocktail containing 100 U of T7 RNA polymerase, 6.7 mM of each NTP, 1 μg of annealed product, and nuclease-free water to complete the volume. After incubation at 37°C for 12 h, 30 μL of nuclease-free water was added to the product, followed by 2 μL of DNase I. The mixture was again incubated to digest the DNA template at 37°C for 15 min. The purification of crRNA was then performed using NucAway^™^ Spin Columns (Thermo Fisher, Waltham, MA, United States) according to the manufacturer’s instructions.

### 2.5. *LwaCas13a* collateral cleavage assay

For *Lwa*Cas13a detection using fluorescence equipment, the 50 μL detection sample contained a detection buffer (60 mM NaCl, 6 mM MgCl_2_, 40 mM Tris-HCl, pH 7.3), 45 nM of purified *Lwa*Cas13a protein, 22.5 nM of crRNA, 0.5 mM of NTP mix, 30 U of T7 RNA polymerase, 125 nM of RNase reporter, 80 U of RNase inhibitor, and 20 μL of the RPA product. The reaction was conducted on a QuantStudio 3 system (Thermo Fisher, Waltham, MA, United States) at 37°C, and the fluorescence values (under 485 nm excitation and 520 nm emission wavelength) were measured every 5 min for up to 2.5 h.

For *Lwa*Cas13a detection with lateral flow-based readout, the synthesized RNA labeled with FAM and biotin at the 5′ and 3′ ends (FAM-UUUUUUUUUUUUUU-Bio, Sangon Biotech, Shanghai, China) (Myhrvold et al., 2018) was used as a reporter RNA at a final concentration of 1 μM in 50 μL of detection mix. Samples were incubated at 37°C for 20 min and then were diluted with HybriDetect Assay Buffer (Milenia Biotec, Geissen, Germany) at a ratio of 1:4. Following this, the solution was added to a HybriDetect 1 lateral flow strip (Milenia Biotec, Geissen, Germany). After incubation for 5 min, photographs of the strips were captured using a camera.

### 2.6. Quantitative real-time polymerase chain reaction assay

Quantitative real-time polymerase chain reaction (qRT-PCR) was performed using the AIV real-time RT-PCR Test Kit (Shenzhen Combined Biotech Co., Ltd. Shenzhen, China) in a 25 μL reaction volume, including 4 μL sample RNA, 21 μL RT-PCR buffer A, and 1 μL RT-PCR buffer B. qRT-PCR cycling conditions were as follows: reverse transcription (RT) at 42°C for 20 min, initial denaturation at 95°C for 10 min, 40 cycles with a denaturing step at 95°C for 15 s, and annealing and elongation steps at 55°C for 30 s. The fluorescence signals were collected after each annealing and elongation step.

### 2.7. Heating unextracted diagnostic samples to obliterate nucleases (HUDSON) protocols

HUDSON treatment was performed as previously described (Myhrvold et al., 2018). Briefly, tissue samples or viral seedstock were mixed with tris (2-carboxyethyl) phosphine (TCEP, at a final concentration of 100 mM) and ethylene diamine tetraacetic acid (EDTA, at a final concentration of 1 mM). The mixture was incubated at 50°C for 20 min, followed by 5 min at 95°C to inactivate nucleases. The treated samples were then subjected to further RPA amplification and *Lwa*Cas13a collateral cleavage assay.

### 2.8. Single-step optimization of CRISPR-Cas13a-based detection

Using the detection method developed above, RPA and CRISPR-Cas13a were detected in two steps. To further simplify the procedure while reducing the risk of contamination, we performed RT, RPA, and CRISPR-Cas13a detection in a single step. The reaction mix consisted of detection buffer, 29.5 μL of Primer Free Rehydration buffer, 100 U of RNase inhibitor, 200 U SuperScrip IV reverse transcriptase (Thermo Fisher, Waltham, MA, United States), 10 U of RNase H (NEB, Ipswich, MA, United States), 2.4 μL each of forward or reverse RPA primers, 45 nM of purified *Lwa*Cas13a protein, 22.5 nM of crRNA, 125 nM RNase reporter, 100 U of T7 RNA polymerase, 5 μL of RNA, and 8 mM of NTP mix, which were used to resuspend the freeze-dried reaction pellets. Thereafter, 5 μL of 280 mM MgOAc was added to complete the reaction. Fluorescence kinetics and lateral-flow-based readouts were collected as described above.

### 2.9. Statistical analysis

Statistical analysis was evaluated by a one-way analysis of variance (ANOVA) test using the GraphPad Prism software (version 8.0, Graph Pad Software Inc., CA, United States). A difference with a value of *p* < 0.01 was considered highly statistically significant. The asterisk indicates the statistical significance: ***p < 0.001.

## 3. Results

### 3.1. Establishment of CRISPR-Cas13a-based detection of AIV

**Figure 2:**
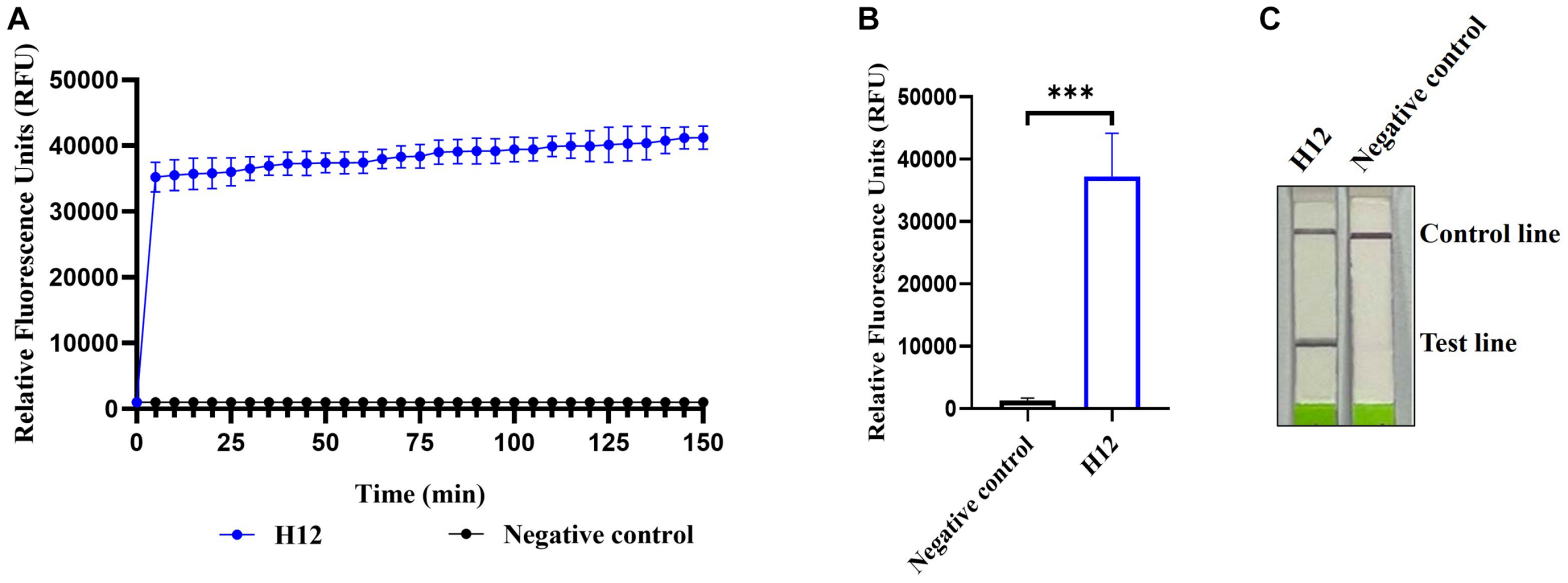
Establishment of CRISPR-Cas13a-based detection of AIV. Fluorescent kinetics **(A)** and fluorescence values **(B)** of CRISPR-Cas13a-based detection of the H12 subtype of AIV. The negative control used nuclease-free water as a template. ***p < 0.001. The error bars indicate standard deviations. **(C)** Lateral flow-based readouts of CRISPR-Cas13a-based detection of the H12 subtype of AIV. The negative control used nuclease-free water as a template.

Among the four pairs of primers for the RPA assay of AIV, the amplification of RPA-F/RPA-R with cDNA of the H12 subtype as a template generated a 211 base pair product that showed as a single band on an agarose gel, whereas no band was observed in the negative control (nuclease-free water was used as the template). Therefore, the RPA-F/RPA-R primer pair was selected for further experiments. In *Lwa*Cas13a collateral cleavage detection, fluorescence kinetics revealed that the background-subtracted fluorescence increased rapidly after 5 min of the reaction, whereas no fluorescent signal was detected in the negative control (Figures 2A,B). For the lateral flow-based readout, the lateral flow strip of H12 appeared as a positive band (Figure 2C). These results indicated the establishment of CRISPR-Cas13a-based detection of the H12 subtype virus.

**Figure 3:**
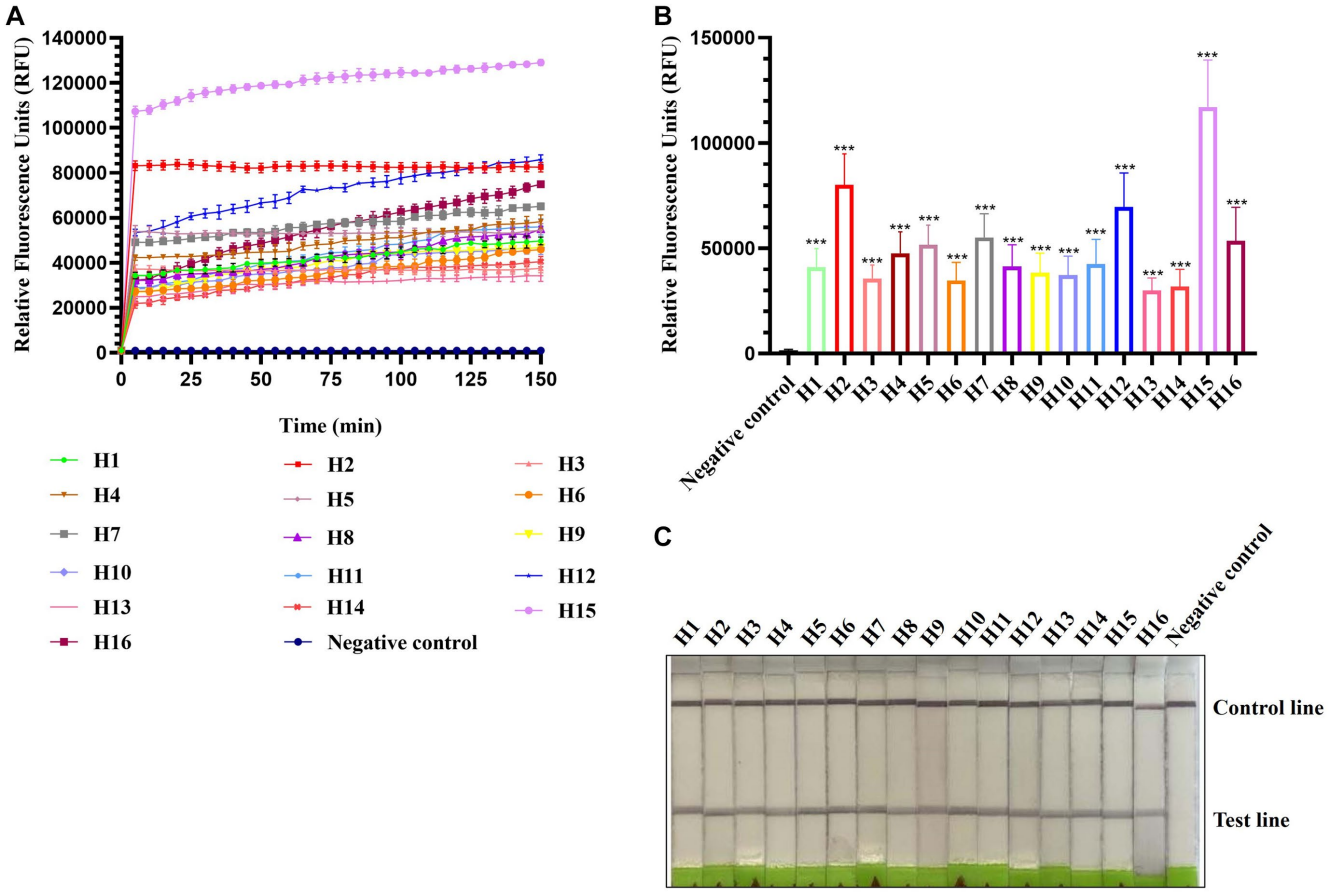
Estimation of CRISPR-Cas13a-based detection of H1–H16 subtypes of AIV. Fluorescent kinetics **(A)** and fluorescence values **(B)** of CRISPR-Cas13a-based detection of H1–H16 subtypes of AIV. The negative control used nuclease-free water as a template. ***p < 0.001. The error bars indicate standard deviations. **(C)** Lateral flow-based readouts of CRISPR-Cas13a-based detection of H1–H16 subtypes of AIV. The negative control used nuclease-free water as a template.

To evaluate the applicability of CRISPR-Cas13a-based detection for other AIV subtypes, H1–H16 subtype viruses were subjected to RPA amplification and CRISPR-Cas13a detection. As shown in Figures 3A,B, all 16 AIV subtypes exhibited evident fluorescent signal increments after 5 min. The lateral flow-based readout also demonstrated positive bands for all subtypes (Figure 3C). These results suggested that the established detection method could be used to detect these 16 AIV subtypes. Notably, the fluorescence signals of each subtype were not identical, possibly because of their different RNA concentrations, which was verified using qRT-PCR (data not shown).

### 3.2. Specificity of CRISPR-Cas13a-based detection of AIV

**Figure 4:**
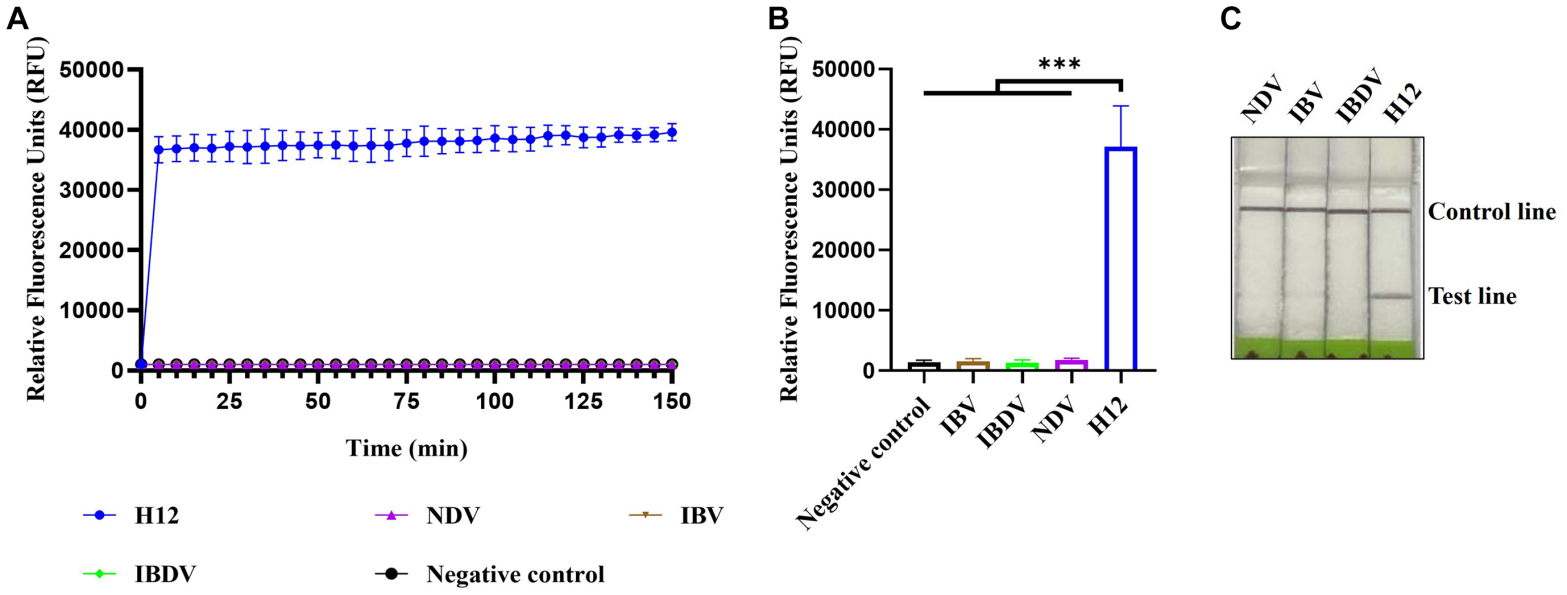
Specificity of CRISPR-Cas13a-based detection of AIV. Fluorescent kinetics **(A)** and fluorescence values **(B)** of CRISPR-Cas13a-based detection of AIV in detecting different avian disease viruses. The negative control used nuclease-free water as a template. ***p < 0.001. The error bars indicate standard deviations. **(C)** Lateral flow-based readout of CRISPR-Cas13a-based detection of AIV in detecting different avian disease viruses.

The specificity of CRISPR-Cas13a-based detection of AIV was analyzed using three different avian viruses (NDV, IBV, and IBDV). The fluorescence kinetics showed that AIV alone produced fluorescence signals, whereas all other samples did not exhibit fluorescence (Figures 4A,B). The results of lateral flow detection were consistent with those of the fluorescence readouts (Figure 4C). These data demonstrate that the established CRISPR-Cas13a-based detection method was highly specific for detecting AIV.

### 3.3. Sensitivity of CRISPR-Cas13a-based detection of AIV

**Figure 5:**
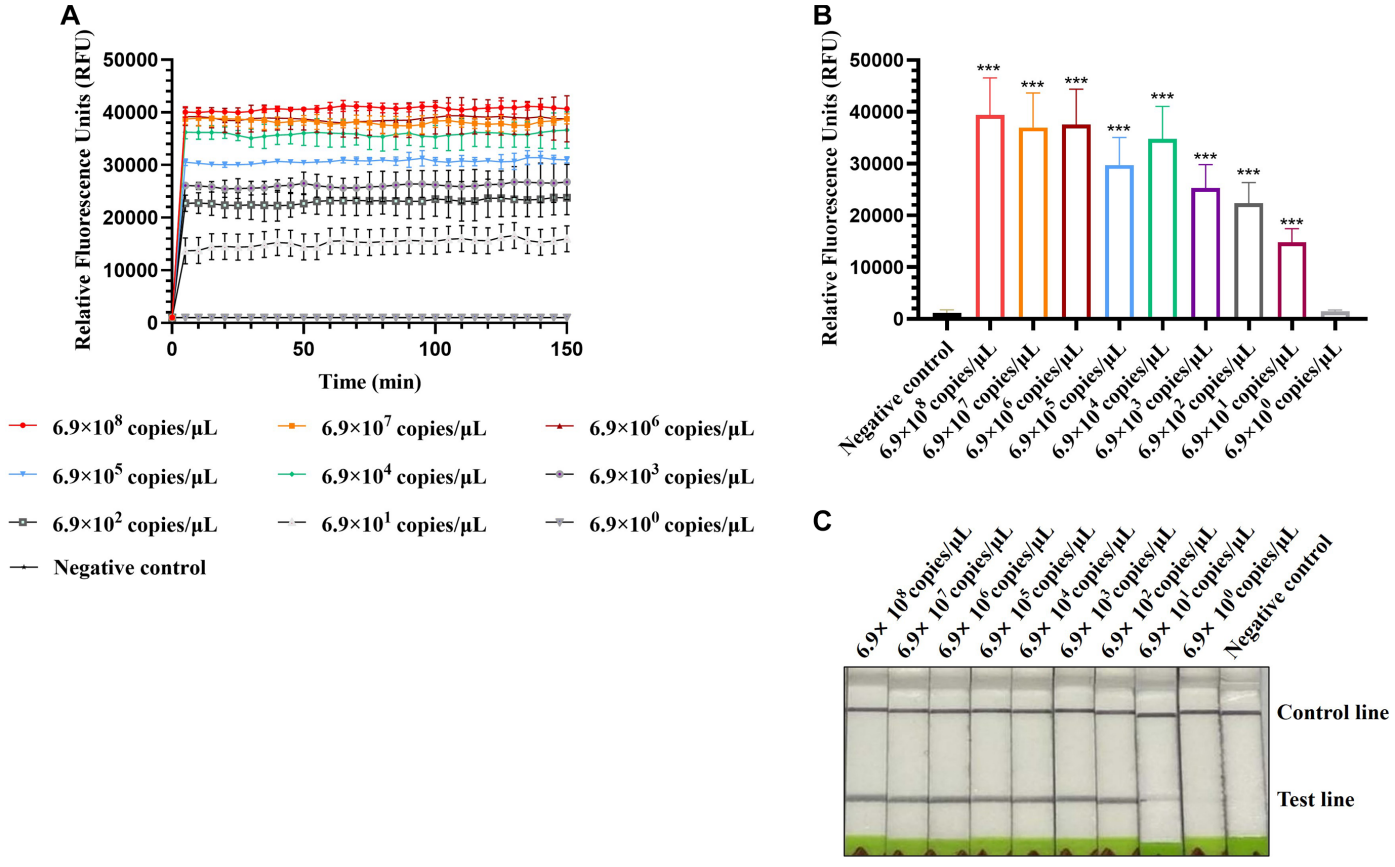
Sensitivity of CRISPR-Cas13a-based detection of AIV. Fluorescent kinetics **(A)** and fluorescence values **(B)** of CRISPR-Cas13a-based detection of AIV in detecting 10-fold dilutions of the H12 subtype of AIV. The negative control used nuclease-free water as a template.***p < 0.001. The error bars indicate standard deviations. **(C)** Lateral flow-based readouts of CRISPR-Cas13a-based detection of AIV in 10-fold dilutions of the H12 subtype of AIV. The negative control used nuclease-free water as a template.

To evaluate the sensitivity of CRISPR-Cas13a-based detection of AIV, 10-fold serial dilutions from 10^−1^ to 10^−10^ of H12 samples were tested. The initial concentration of H12 cDNA was 219.1 μg/μL containing 6.9 × 10^10^ copies/μL. The fluorescent signal could be detected in the 10^−9^ dilution, which indicated that the limit of detection using fluorescent readout was below 6.9 × 10^1^ copies/μL (Figures 5A,B). In the lateral flow-based readout, a positive band was detected on the strip containing the 10^−8^ dilution, demonstrating a detection limit as low as 6.9 × 10^2^ copies/μL for H12 cDNA (Figure 5C).

### 3.4. Repeatability and reproducibility of CRISPR-Cas13a-based detection of AIV

The repeated tests within and between batches were performed using three concentrations: 6.9 × 10^8^ copies/μL (high concentration), 6.9 × 10^5^ copies/μL (medium concentration), 6.9 × 10^2^ copies/μL (low concentration) of AIV nucleic acids. The intra-batch tests were performed in three repeats with one run, while the inter-batch analyses were conducted by three independent runs within 3 days. The coefficient of variation (CV) of repeated tests intra-batch and inter-batch was determined by calculating the fluorescence values. As shown in Table 3, the CVs of within and between batches were less than 3% and 4%, respectively, suggesting that the established method was well stable.

**Table 3:** Results of repeated tests within and between batches of CRISPR-Cas13a-based detection.

AIV nucleic acid concentration (copies/μL)	Repeatability (Intra-batch test)			Reproducibility (Inter-batch test)		
	Mean	SD	CV	Mean	SD	CV
High (6.9 × 10^8^)	40695.00	480.90	1.12%	39788.99	692.28	1.74%
Medium (6.9 × 10^5^)	30684.95	519.59	1.70%	31720.13	487.02	1.53%
Low (6.9 × 10^2^)	23065.35	573.24	2.49%	24895.62	983.60	3.95%

Mean, average fluorescence values of three independent reactions; SD, standard deviation; CV, coefficient of variation.

### 3.5. Comparison of CRISPR-Cas13a-based detection with qRT-PCR

Thirty clinical samples were tested using qRT-PCR (Beijing Scenk Biotechnology Development Co., Ltd., Beijing, China) and CRISPR-Cas13a-based detection in parallel. For the qRT-PCR assay, 22 samples were found AIV-positive, whereas eight samples were negative. The CRISPR-Cas13a-based detection showed perfect concordance with the qRT-PCR results (Table 4).

**Table 4:** Detection in clinical samples via CRISPR-Cas13a-based detection and qRT-PCR assay.

Assay	Number of samples	
	Positive	Negative
CRISPR-Cas13a-based detection	22	8
qRT-PCR	22	8

### 3.6. CRISPR-Cas13a-based detection of AIV without nucleic acid extraction

**Figure 6:**
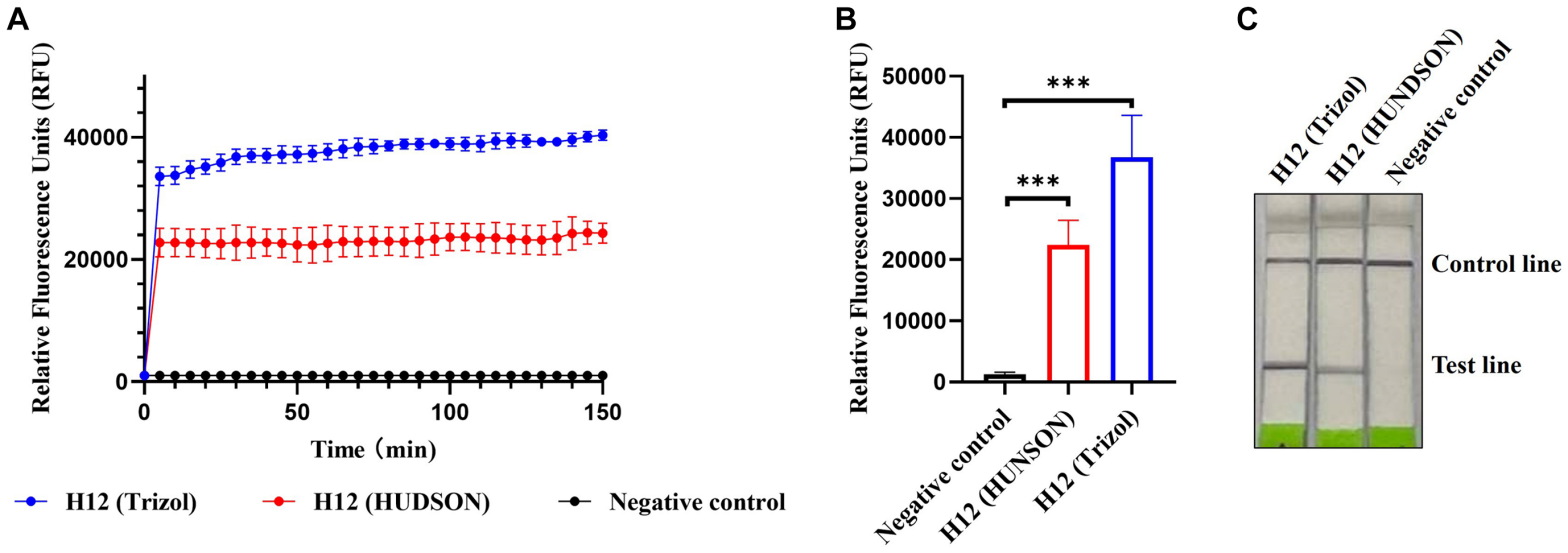
CRISPR-Cas13a-based detection of AIV without nucleic acid extraction. Fluorescent kinetics **(A)** and fluorescence values **(B)** of CRISPR-Cas13a-based detection of AIV without nucleic acid extraction in detecting the H12 subtype of AIV. The negative control used nuclease-free water as a template. ***p < 0.001. The error bars indicate standard deviations. **(C)** Lateral flow-based readouts of CRISPR-Cas13a-based detection of AIV without nucleic acid extraction in detecting the H12 subtype of AIV. The negative control used nuclease-free water as a template.

Considering the application of the platform in the field, we introduced HUDSON treatments to simplify the procedure of nucleic acid extraction and evaluated its compatibility with RPA amplification and Cas13a-based detection. As shown in Figures 6A,B, the fluorescent signals of HUDSON treated samples were much higher than those of the control but slightly lower than those of nucleic acid extracted samples. The lateral flow-based readout showed that lateral flow strips of HUDSON-treated samples also appeared as positive bands (Figure 6C).

### 3.7. Single-step optimization of CRISPR-Cas13a-based detection of AIV

**Figure 7:**
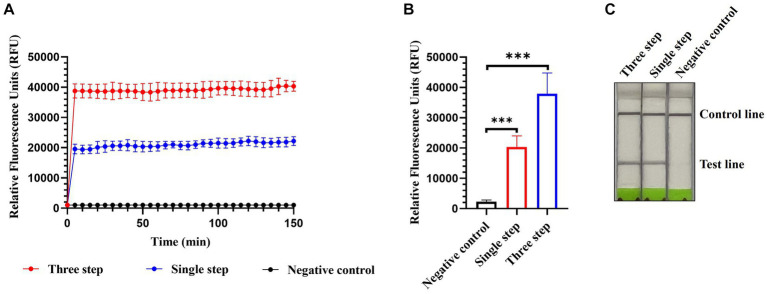
Single-step optimization of CRISPR-Cas13a-based detection of AIV. Fluorescent kinetics **(A)** and fluorescence values **(B)** of single-step optimization of CRISPR-Cas13a-based detection of the H12 subtype of AIV. The negative control used nuclease-free water as a template. ***p < 0.001. The error bars indicate standard deviations. **(C)** Lateral flow-based readouts of single-step optimization of CRISPR-Cas13a-based detection of the H12 subtype of AIV. The negative control used nuclease-free water as a template.

Single-step optimization of the detection method was performed by combining RT, RPA, and CRISPR-Cas13a-based detection into a single step. SuperScript IV Reverse Transcriptase and RNase H were added to the reaction mix to increase the efficiency of reverse transcription. Both the fluorescence and lateral flow-based readouts demonstrated that the optimized single-step CRISPR-Cas13a-based method could detect AIV (Figures 7A–C) but with weaker signals relative to the previously established three-step assay using fluorescence readouts (Figures 7A,B).

## 4. Discussion

Viral infectious diseases, particularly the emerging and re-emerging ones, are a major threat to human and animal health (Unnewehr et al., 2018). Coronavirus disease 2019, which emerged in late 2019, rapidly spread globally, seriously affecting human health and life systems and causing severe negative impacts on the global economy (Wang et al., 2020). The first outbreak of African swine fever (ASF) in China in August 2018 also caused huge losses to the swine industry (Wen et al., 2019); ASF remains prevalent in many countries, threatening global trade (Han et al., 2020). Rapid, sensitive, and accurate diagnosis of viral infectious diseases is imperative for controlling the incidence of epidemics, reducing the risk of infection, and protecting humans and animals. At present, the most commonly used nucleic acid detection method is qRT-PCR (Watzinger et al., 2006), which has good specificity and high sensitivity. However, this method requires specialized personnel, expensive machines, and a relatively long time, thus limiting its application in the field. An ideal diagnostic method for viral infectious diseases should be sensitive, specific, rapid, low-cost, simple to operate, and require minimal or no instrumentation. To date, no method has met all these requirements. Therefore, more effective molecular diagnostic methods need to be developed.

Recently, CRISPR-Cas systems have not only been developed as powerful gene-editing tools but have also exhibited their unique advantages in diagnosing infectious diseases (Kaminski et al., 2021; Mustafa and Makhawi, 2021). Currently, CRISPR-Cas9, CRISPR-Cas12, and CRISPR-Cas13 are the most well-studied and widely used CRISPR-Cas systems (Koonin et al., 2017). Cas9 recognizes and cleaves DNA via a protospacer adjacent motif (PAM, 5′-NGG-3′) (Cong et al., 2013). Cas12 is guided by the T-rich PAM sequences (5′-TTN-3′) of target sequences and exhibits cleavage activity on both DNA and RNA (Makarova et al., 2020). Cas13 cleaves RNA by identifying sequences containing protospacer flanking sites (Abudayyeh et al., 2016). Thus, Cas13 has fewer restrictions on target sequences than the other two effector proteins. Currently, CRISPR-Cas13a has been successfully used for the detection of severe acute respiratory syndrome coronavirus 2 (Fozouni et al., 2021; Zhang et al., 2022), African swine fever virus (Wei et al., 2022), *Mycobacterium tuberculosis* (Bai et al., 2022; Ren et al., 2023), *Salmonella* spp. (An et al., 2021), tomato spotted wilt virus (Zhang et al., 2021), and so on. In this study, a CRISPR-Cas13a-based detection platform for AIV was developed, in which the high sensitivity of RPA amplification and the good specificity of *Lwa*Cas13a collateral cleavage were combined. Both RPA and *Lwa*Cas13a collateral cleavage were performed at 37°C, requiring less equipment than the widely used qRT-PCR assay. The limit of detection was found to be 69 and 690 copies/μL using fluorescence and lateral flow readouts, respectively. In addition to the difference in sensitivity, the fluorescence and lateral flow-based readouts also have tradeoffs between the sample batch size and equipment requirements. For fluorescence readouts, a large number of samples can be analyzed simultaneously but fluorescence detector is required. In contrast, lateral flow-based readouts require a heat block alone to conduct RPA and *Lwa*Cas13a collateral cleavage; however, it cannot test a large number of samples in parallel.

To address diagnostic needs at the grassroots level, two improvements have been made: HUDSON treatment and single-step optimization. For the former, samples were mixed with TCEP/EDTA and heated to lyse viral particles and inactivate ribonucleases, thus simplifying the tedious steps of nucleic acid extraction. In addition to HUDSON treatment, two other methods (including RNAGEM V from MicroGEM and Diff from IFFERENCE) for rapidly obtaining nucleic acids have been tried in parallel. The results revealed that HUDSON treatment was the most suitable for developing point-of-care testing (data not shown). For the single-step optimization, the RT, RPA, and CRISPR-Cas13a collateral cleavage assays were combined into a single step, which not only simplified the operation steps but, more importantly, reduced the risk of contamination. However, the fluorescence signals of the single-step optimization were weaker than those of the established three-step assay, implying that its sensitivity may be reduced and additional advances are required to remedy this.

In the present study, a CRISPR-Cas13a-based detection platform for AIV was established. This novel method could specifically detect AIV RNA with fluorescence and lateral flow-based readouts. HUDSON treatment was further introduced to detect viral RNA without nucleic acid extraction. Single-step optimization was used to perform RT, RPA, and CRISPR-Cas13a detection in a tube. These advances could greatly simplify sample processing and detection procedures and reduce personnel time and contamination risk; however, at the expense of weakened signals. Future research is required toward the development of a platform suitable for point-of-care testing.

## Data availability statement

The original contributions presented in the study are included in the article/supplementary material, further inquiries can be directed to the corresponding authors.

## Ethics statement

The animal study was approved by Animal Experimentation and Laboratory Animal Welfare Committee of Shenyang Agricultural University (No. 2021121001). The study was conducted in accordance with the local legislation and institutional requirements.

## Author contributions

YuW: Writing – original draft, Investigation. JZ: Writing – original draft, Investigation. ZS: Writing – original draft, Data curation. YbL: Writing – original draft, Resources. YiL: Writing – original draft, Data curation. YaL: Writing – original draft, Data curation. YiW: Data curation, Writing – original draft. ZL: Data curation, Writing – original draft. XuW: Writing – review & editing, Conceptualization. XiW: Conceptualization, Writing – review & editing, Funding acquisition.
